# Terminal *N*-Acetylgalactosamine-Specific Leguminous Lectin from *Wisteria japonica* as a Probe for Human Lung Squamous Cell Carcinoma

**DOI:** 10.1371/journal.pone.0083886

**Published:** 2013-12-13

**Authors:** Keisuke Soga, Futaba Teruya, Hiroaki Tateno, Jun Hirabayashi, Kazuo Yamamoto

**Affiliations:** 1 Department of Integrated Biosciences, Graduate School of Frontier Sciences, The University of Tokyo, Kashiwa, Chiba, Japan; 2 Research Center for Medical Glycoscience, Advanced Industrial Science and Technology (AIST), Tsukuba, Ibaraki, Japan; Ghent University, Belgium

## Abstract

*Millettia japonica* was recently reclassified into the genus *Wisteria japonica* based on chloroplast and nuclear DNA sequences. Because the seed of *Wisteria floribunda* expresses leguminous lectins with unique *N*-acetylgalactosamine-binding specificity, we purified lectin from *Wisteria japonica* seeds using ion exchange and gel filtration chromatography. Glycan microarray analysis demonstrated that unlike *Wisteria floribunda* and *Wisteria brachybotrys* lectins, which bind to both terminal *N*-acetylgalactosamine and galactose residues, *Wisteria japonica* lectin (WJA) specifically bound to both α- and β-linked terminal *N*-acetylgalactosamine, but not galactose residues on oligosaccharides and glycoproteins. Further, frontal affinity chromatography using more than 100 2-aminopyridine-labeled and *p*-nitrophenyl-derivatized oligosaccharides demonstrated that the ligands with the highest affinity for *Wisteria japonica* lectin were GalNAcβ1-3GlcNAc and GalNAcβ1-4GlcNAc, with *K*_a_ values of 9.5 × 10^4^ and 1.4 × 10^5^ M^-1^, respectively. In addition, when binding was assessed in a variety of cell lines, *Wisteria japonica* lectin bound specifically to EBC-1 and HEK293 cells while other *Wisteria* lectins bound equally to all of the cell lines tested. *Wisteria japonica* lectin binding to EBC-1 and HEK293 cells was dramatically decreased in the presence of *N*-acetylgalactosamine, but not galactose, mannose, or *N*-acetylglucosamine, and was completely abrogated by β-hexosaminidase-digestion of these cells. These results clearly demonstrate that *Wisteria japonica* lectin binds to terminal *N*-acetylgalactosamine but not galactose. In addition, histochemical analysis of human squamous cell carcinoma tissue sections demonstrated that *Wisteria japonica* lectin specifically bound to differentiated cancer tissues but not normal tissue. This novel binding characteristic of *Wisteria japonica* lectin has the potential to become a powerful tool for clinical applications.

## Introduction

 Many types of lectins, which are defined as proteins having the ability to bind sugars, have been isolated from bacteria, plant and animals. Cell surface glycans and oligosaccharides attached to proteins play important roles in cell-cell interactions and biological functions of the cell and glycoproteins *in vivo*. Glycoproteins in the blood and pathological tissues frequently possess unusual glycan structures. To investigate the significance of distinct glycosylation states, it is crucial to identify lectins that discriminate between unique and strict sugar structures among many kinds of oligosaccharides.

*Wisteria japonica* was first introduced to Europe by Phililpp von Siebold in 1830 together with *Wisteria floribunda* and *Wisteria brachybotrys* [1]. At that time, *Wisteria japonica* had been placed in the genus *Millettia* based on the terminal racemes in *Wisteria* as opposed to lateral racemes in *Millettia* [1] and the absence of a pair of thickened callosities, which differed from other *Wisteria* species [1]. However, recent sequence studies of chloroplast and nuclear DNA have shown that the *Wisteria japonica* belongs in the genus *Wisteria* rather than *Millettia* [1–4]. Wisteria floribunda agglutinin (WFA) has been studied in detail and is reported to have unique biological activities, including hemagglutinating capacity and the induction of lymphocyte activation [5–7]. WFA has a greater affinity for *N*-acetylgalactosaminides than for galactosides [8], and subsequent study demonstrated that it could bind to GalNAcβ1-4GlcNAc [9], whose sugar sequence was first observed on the pituitary glycoprotein hormones, lutropin (LH), thyrotropin (TSH) and follitropin (FSH) [10,11]. However, this sugar structure is the most uncommon constituent of glycans on vertebrate proteins. The GalNAcβ1-4GlcNAc sequence of oligosaccharides is formed by the activity of β1,4-*N*-acetylgalactosaminyltransferases, and such transferases are rarely expressed in distinct tissues and organs [12,13]. Recently, GalNAcβ1-4GlcNAc modification of *N*-glycans has been well documented [14,15]; however, the implication of this novel modification with respect to the posttranslational control of protein function remains unknown because of the lack of a specific probe for the GalNAcβ1-4GlcNAc sequence.

 In the present study, we purified a novel leguminous lectin from *Wisteria japonica* seeds whose specificity is specific for GalNAcβ1-4GlcNAc and GalNAcβ1-3GlcNAc sequences. Interestingly, the *Wisteria japonica* lectin (WJA) strongly bound to EBC-1 human squamous cell carcinoma cells and specifically stained the cancerous portions of lung specimens from lung squamous cell carcinoma patients.

## Materials and Methods

### Preparation of *Wisteria lectins*

*Wisteria* seeds (*Wisteria floribunda, Wisteria brachybotrys*, and *Wisteria japonica*) were purchased from Exotic Plants Co. (Tateyama, Japan) and classification of the seeds were confirmed by the help of Dr. Jin Murata (Koishikawa Botanical Gardens, The University of Tokyo). Purification of *Wisteria* lectins was performed according to the method of Toyoshima et al. [5] with minor modifications. Briefly, finely powdered *Wisteria* seeds were suspended in 10 mM phosphate buffer (pH 7.4) containing 0.15 M NaCl (PBS) and stirred at 4°C for 18 h. After centrifugation at 17,000 × *g* for 1 h, clear supernatant was combined with (NH_4_)_2_SO_4_ to give 80% saturation. The precipitated fraction was obtained by centrifugation, resuspended in distilled water and dialyzed against 50 mM phosphate buffer (pH 5.0). Lectin fractions were purified by cationic ion exchange chromatography on a Toyopearl SP-550C column (Toso, Tokyo, Japan) followed by gel filtration chromatography on a HiLoad 26/60 Superdex 200 column (prep grade, GE Healthcare, Buckinghamshire, UK) using the AKTA Explorer system (GE Healthcare). The activity of lectin was monitored by hemagglutination using sialidase (Nacalai Tesque, Kyoto, Japan)-treated mouse red blood cells. The purity of the lectin was checked by SDS polyacrylamide gel electrophoresis according to the method of Laemmli. Purified lectin fractions were dialyzed against distilled water and lyophilized. The N-terminal amino acid sequences of the purified lectins were analyzed by a Procise 492cLC protein sequencer (Applied Biosystems, Foster City, CA).

### Glycan microarray

 The sugar-binding specificity of *Wisteria* lectins was analyzed by the glycan microarray described in detail in Figure S1 [16]. *Wisteria* lectins were labeled with Cy3-*N*-hydroxysuccinimide ester (NHS-Cy3, GE Healthcare) as described previously [16]. After removing excess amounts of NHS-Cy3 by gel filtration on a Sephadex G-25 column (GE Healthcare), Cy3-labeled *Wisteria* lectins (5 µg/ml) in a probing buffer [25 mM Tris-HCl (pH 7.4), 0.15 M NaCl, 1% (v/v) Triton-X100, 1 mM MnCl_2_, 1 mM CaCl_2_] were applied to each chamber of a glass slide (100 µl/well) and incubated at 20°C for 18 h. After washing twice with probing buffer, the binding of lectins to the glycoconjugate microarray was detected using an evanescent field-activated fluorescence scanner, GlycoStation Reader 1200 (GlycoTechnica, Hokkaido, Japan) in Cy3 mode.

### Frontal affinity chromatography (FAC)

 WJA was coupled to NHS-activated Sepharose (GE Healthcare) at a concentration of 9.0 mg/ml according to the manufacturer's protocol. WJA-Sepharose was suspended in 10 mM Tris-HCl (pH 7.6) containing 0.15 M NaCl (TBS) and then packed into a miniature column (2 mm × 10 mm). FAC was performed using an automated system (FAC-1), as described previously [17,18]. Briefly, the WJA-Sepharose column was slotted into a stainless holder and then connected to the FAC-1 machine. Flow rate and column temperature were kept at 0.125 ml/min and 25°C, respectively. After equilibration with TBS, additional volumes (0.5–0.8 ml) of 2-aminopyridine (PA)-labeled glycans or *p*-nitrophenyl (*p*NP)-derivatized glycans (3.7–7.5 μM) were successively injected into the column by an auto-sampling system. Elution of PA-glycans was monitored by fluorescence at an excitation wavelength of 310 nm and an emission wavelength of 380 nm. The elution front relative to that of PA-labeled Manα1-3Manβ1-4GlcNAcβ1-4GlcNAc, i.e., V-V_0_, was then determined. The association constants (*K*_a_) were obtained from the V-V_0_ and B_t_ according to the FAC equation, V-V_0_ = B_t_ × *K*_a_ [17,18]. In case of *p*NP-derivatized glycans, glycans were monitored at 280 nm and the elution front relative to that of *p*NP-α-fucose was measured.

### Lectin staining of the cells

 Daudi, EBC-1, HL60, HeLaS3, K562 and SW480 cells were obtained from the Cell Resource Center for Biochemical Research (Tohoku University, Miyagi, Japan) and maintained in RPMI 1640 medium (Sigma-Aldrich, St. Louis, MO) supplemented with 10% heat-inactivated fetal calf serum (FCS), 100 µg/ml penicillin, 100 U/ml streptomycin, 2 mM glutamine, and 50 µM 2-mercaptoethanol under 5% CO_2_ at 37°C. HEK293 and HeLa cells (Cell Resource Center for Biochemical Research) were cultured in Dulbecco's modified Eagle's medium supplemented with 10% heat-inactivated FCS, 100 µg/ml penicillin, 100 U/ml streptomycin, 2 mM glutamine, and 50 µM 2-mercaptoethanol under 5% CO_2_ at 37°C. To modify cell surface glycans, EBC-1 or HEK293 cells suspended in piperazine-1,4-bis(2-ethanesulfonic acid)-NaOH (pH 6.0) containing 0.15 M NaCl, 0.1% bovine serum albumin (BSA) and 0.1% NaN_3_ were exposed to β-*N*-acetylhexosaminidase from *Xanthomonas maninotis* (New England Biolabs, Ipswich, MA) at 37°C for 1 h, and the binding of biotinylated WJA, WFA or WBA was then measured using flow cytometry. Briefly, 1 × 10^5^ cells in Hanks' balanced salt solution (HBSS) containing 0.35 mg/ml NaHCO_3_, 0.1% BSA and 0.1% NaN_3_ was incubated at 4°C for 30 min with 1 µg/ml of each biotinylated lectin in the presence of 25 mM galactose (Gal), mannose, glucose, *N*-acetylgalactosamine (GalNAc), or *N*-acetylglucosamine (GlcNAc). After washing twice with HBSS, the cells were incubated with 1 µg/ml of *R*-phycoerythrin (PE)-labeled streptavidin (BioLegend, San Diego, CA) on ice for 30 min. After the cells were washed with HBSS and suspended in HBSS containing 1 µg/ml propidium iodide (PI), the fluorescence of the stained cells was measured using a FACS Calibur and CellQuest software (BD Biosystems, San Jose, CA). The fluorescence at 575 nm associated with PE on the surface of the cells was recorded and converted to a mean fluorescence intensity (MFI). In total, 10,000 events gated by forward and side scattering and exclusion of PI were acquired for analysis.

### Lectin histochemistry

 WJA staining was performed using biotinylated WJA. Human normal and cancerous lung tissue sections were purchased from Shanghai Outdo Biotech Co. (Shanghai, China). After the tissue sections were deparaffinized, endogenous peroxidase was blocked by incubating the sections with PBS containing 3% hydrogen peroxide. The sections were then blocked with PBS containing 0.5% BSA for 30 min at room temperature, followed by incubation with 2.5 µg/ml of biotinylated WJA in PBS at 4°C for 18 h. After washing the sections twice with PBS, the sections were incubated with avidin-biotin-horseradish peroxidase complex using Vectastain Elite ABC kit (Vector Laboratories, Burlingame, CA), washed twice with PBS and reacted with 3,3’-diaminobenzidine tetrahydrochloride (DAB) for visualization. The developed slides were washed four times with PBS and counterstained with hematoxylin. After washing with water, the sections were dehydrated and mounted. Sections were observed under a BX60 microscope equipped with a DP-71 digital camera system (Olympus, Tokyo, Japan). WJA staining in the presence of 200 mM GalNAc was also performed as a negative control. Experiments using human materials were conducted in accordance with a comprehensive, high quality care program, which has been approved by the Life Science Research Committee of the Graduate School of Frontier Sciences of The University of Tokyo guided by the Life Science Committee of The University of Tokyo.

## Results

### Purification of *Wisteria lectins*

**Figure 1:**
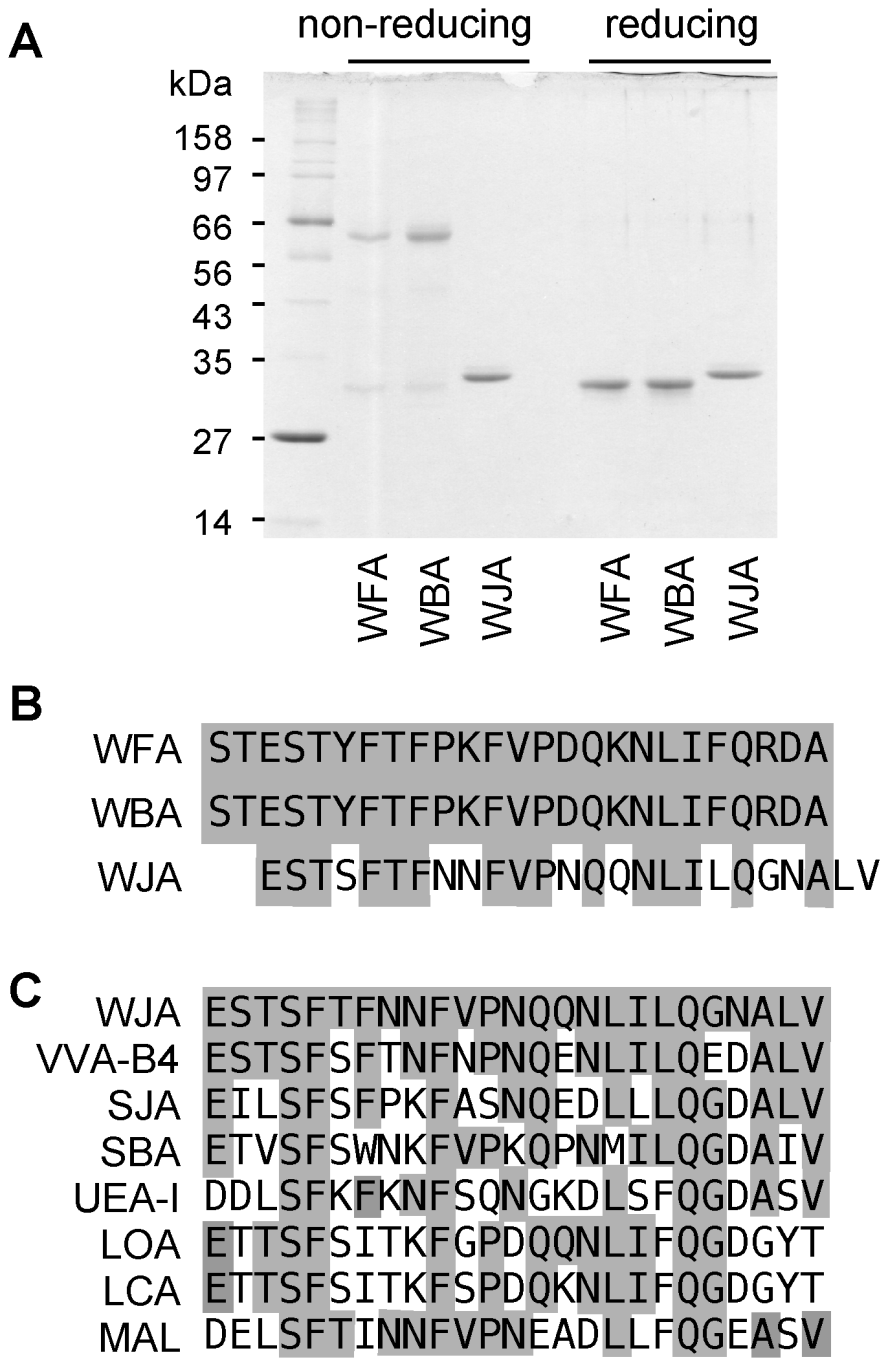
Characterization of *Wisteria* lectins. (A) SDS polyacrylamide gel electrophoresis of purified *Wisteria* *floribunda* (WFA), *Wisteria* *brachybotrys* (WBA) and *Wisteria* *japonica* (WJA) lectins under non-reducing and reducing conditions. (B) N-terminal amino acid sequences of WFA, WBA and WJA. Identical amino acid residues are highlighted. (C) N-terminal amino acid homology between WJA and other leguminous lectins. Identical amino acid residues are highlighted. *Vicia* *virosa* isolectin B4 (VVA-B4), *Sophra* *japonica* lectin (SJA), soybean lectin (SBA), *Ulex* *europeus* lectin-I (UEA-I), *Lachrus* *ochrus* lectin (LOA), *Lens* *curinaris* lectin (LCA), and *Maackia* *amurensis* leukoagglutinin (MAL).

 Ammonium sulfate fractions extracted from *Wisteria japonica, Wisteria floribunda* and *Wisteria brachybotrys* seeds were applied to an SP-Toyopearl 550C column and fractions with hemagglutinating activities were immediately eluted from *Wisteria floribunda* and *Wisteria brachybotrys* extracts when the buffer was changed to phosphate buffer containing 500 mM NaCl, as reported previously [5]. By contrast, hemagglutinating activity was recovered in the flow through fraction from *Wisteria japonica* with more than 80% purity. These flow through fractions were then subjected to gel filtration on a HiLoad 26/60 Superdex 200 column and the fraction with hemagglutinating activity was detected as a single peak corresponding to 120 kDa. Purified *Wisteria japonica* lectin (WJA) appeared as a single band with a relative molecular weight of 30 kDa on SDS-PAGE under both reducing and non-reducing conditions (Figure 1A). *Wisteria floribunda* lectin (WFA) and *Wisteria brachybotrys* lectin (WBA) were also purified on a HiLoad 26/60 Superdex 200 column as single bands with relative molecular weights of 28 kDa and 56 kDa on SDS-PAGE under reducing and non-reducing conditions, respectively (Figure 1A). The yields of WJA, WFA and WBA purified from 100 g of individual seeds were approximately 200, 100 and 100 mg, respectively. The N-terminal amino acid sequences of WJA, WFA and WBA are shown in Figure 1B. The 25 N-terminal amino acid residues of WFA and WBA were identical, while some of the N-terminal residues of WJA differed. Based on protein BLAST homology search, WJA is highly homologous to GalNAc-binding *Vicia villosa* lectin (VVA-B4), *Sophora japonica* lectin (SJA), soybean lectin (SBA), fucose-binding *Ulex europaeus* lectin-I (UEA-I), mannose-binding *Lens culinaris* (LCA) and *Lathyrus ochrus* lectins (LOA), and sialylgalactose-binding *Maackia amurensis* leukoagglutinin (MAL) (Figure 1C).

### Glycan microarray analysis of *Wisteria lectins*

**Figure 2:**
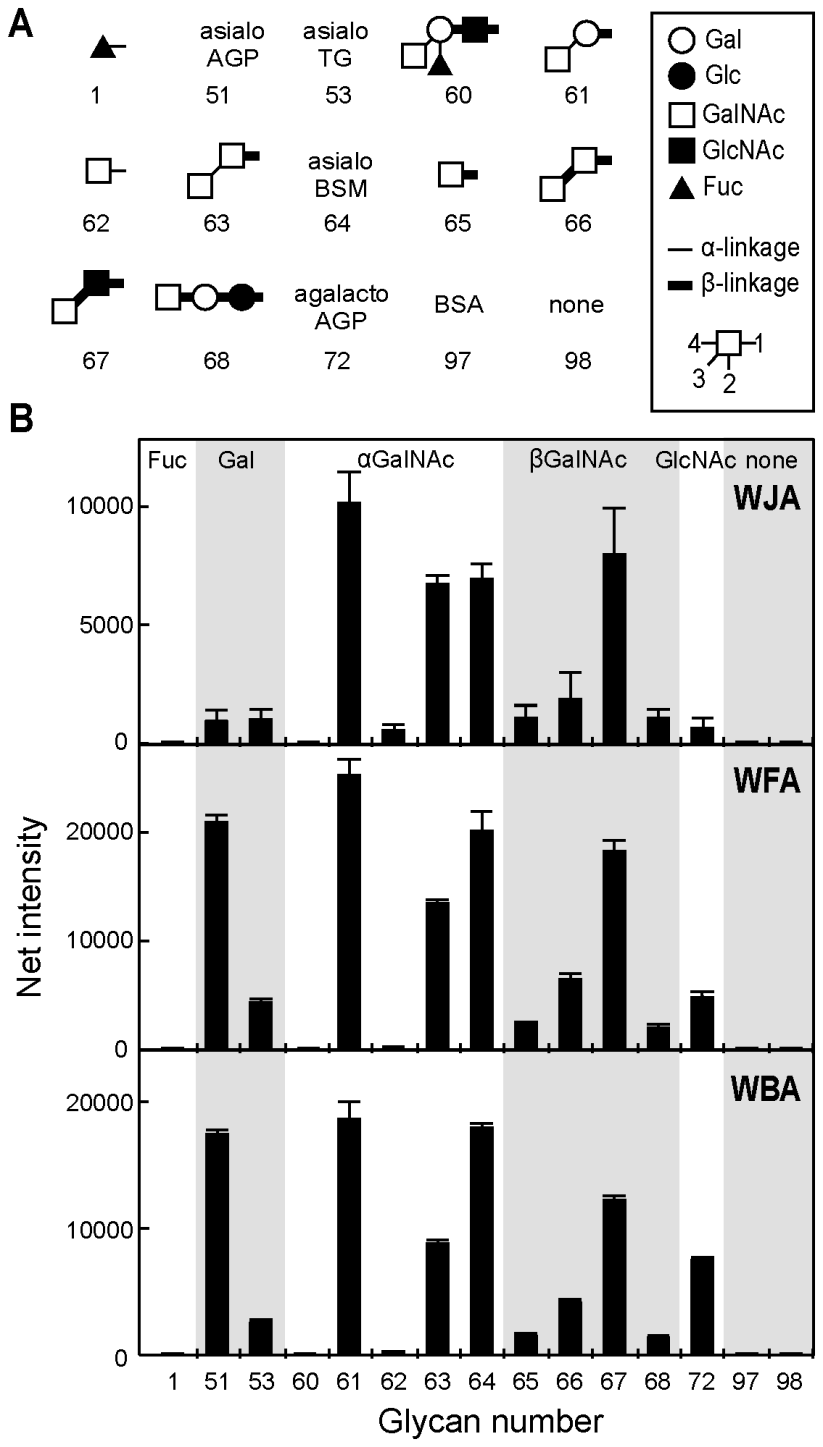
Binding activity of Wisteria lectins to immobilized multivalent oligosaccharides and glycoproteins by glycan array analysis. (A) Structures of polyacrylamide-based oligosaccharides and glycoproteins used for glycan array analysis. All oligosaccharides and glycoproteins used in this experiment (98 in total) are shown in Figure S1. Symbols corresponding to each monosaccharide are shown in the panel. Thin and thick bars represent alpha- and beta-linkages, respectively. Glycosidic linkage positions are shown by the numbers on the lower side of the panel. (B) Binding of each *Wisteria* lectin measured by an evanescent field-activated fluorescence scanner is shown. Oligosaccharides and glycoproteins are classified based on terminal sugar residues and indicated at the top of the upper panel. The data shown are the mean ± SD from three independent spots.

 To compare the sugar-binding specificities of *Wisteria* lectins, Cy3-labeled lectins were subjected to glycan microarray with a variety of polyacrylamide-derivatized oligosaccharide polymers and glycoproteins (Figure S1). WFA is widely used as a GalNAcβ1-4GlcNAc-specific probe [9,19–21] since a lectin with GalNAcβ1-4GlcNAc-binding specificity is unique among lectins, whose specificities are precisely determined [22]. Actually, microarray data showed that WFA bound to α- and β-linked terminal GalNAc-containing oligosaccharides, including GalNAcβ1-4GlcNAc (Figure 2 and Figure S2). Approximately 70% of total sugar moieties from bovine submaxillary mucin (BSM) are NeuAcα2-6GalNAc [23], thus asialo BSM, which contains predominantly GalNAc residues, was a good ligand for WFA. Further, asialo and agalacto α1-acid glycoprotein (AGP) and asialo thyroglobulin (TG) were also good ligands for WFA, indicating that WFA could also bind to terminal Gal residues. WFA bound to agalactosylated α1-acid glycoprotein (Figure 2, glycan 72). This protein has tetraantennary complex-type glycans, indicating that terminal GlcNAc having the sequence GlcNAcβ1-2Man, GlcNAcβ1-4Man, or GlcNAcβ1-6Man may be the ligand of this lectin. WBA showed the same sugar binding specificity as WFA (Figure S2). By contrast, WJA bound to oligosaccharides and glycoproteins possessing non-reducing α- and β-linked GalNAc residues, but not Gal, without exception (Figure 2B).

### Frontal affinity chromatography of WJA

**Figure 3:**
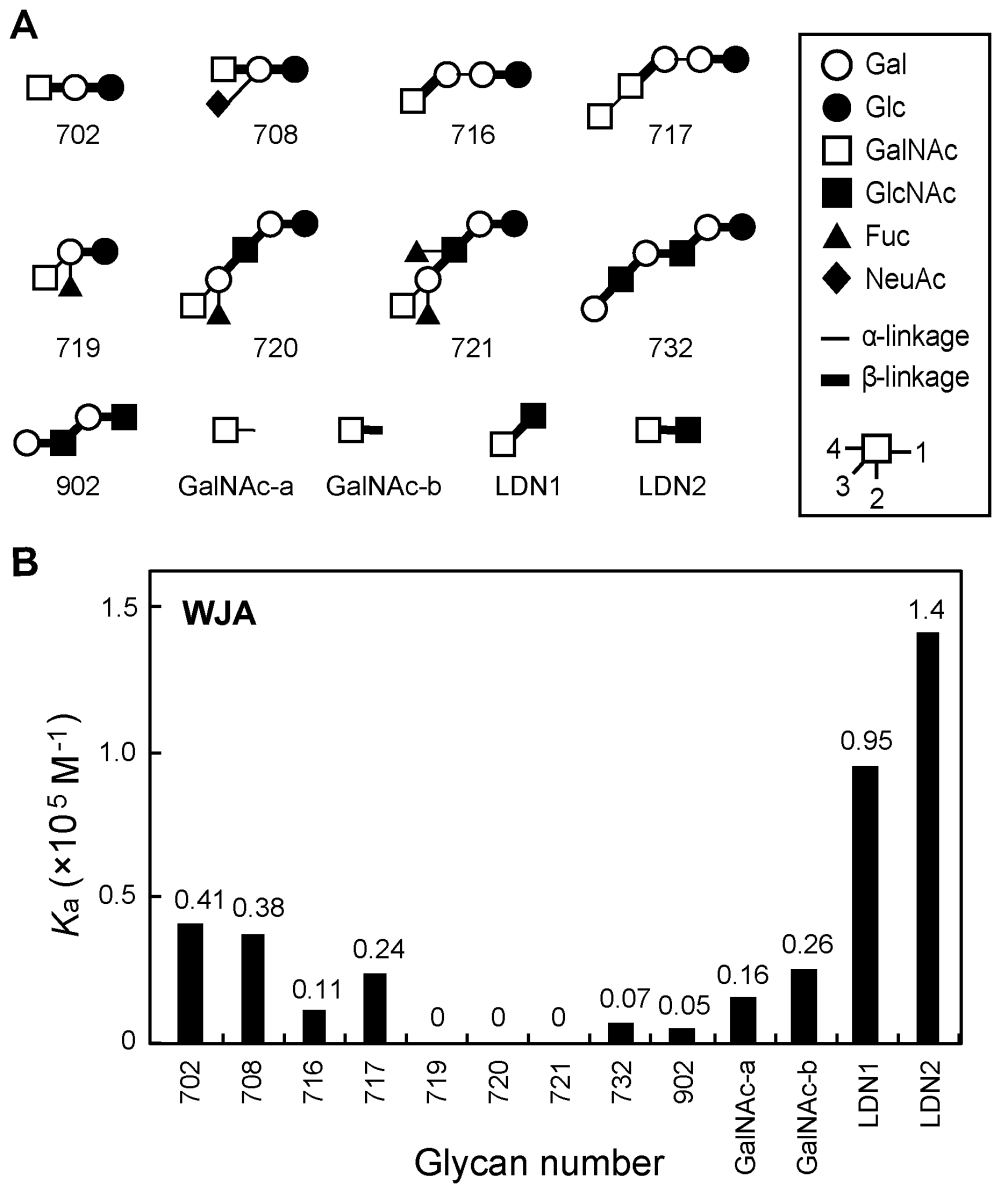
Frontal affinity chromatography (FAC) analysis of WJA with PA-labeled and *p*NP-derivatized oligosaccharides. (A) Structures of PA-labeled and *p*NP-derivatized oligosaccharides used for FAC. All of the oligosaccharides used in this experiment (130 total) are shown in Figure S3. (B) The *K*_a_ value of each oligosaccharide for WJA was calculated as described in Materials and Methods. The *K*_a_ values represent the results of three independent experiments.

 To determine the precise sugar-binding specificity of WJA, FAC was performed using more than 100 different PA-labeled glycans and *p*NP-derivatized glycans (Figure S3). As shown in Figure 3 and Figure S4, WJA bound specifically to GalNAcβ1-3GlcNAc (LDN1) and GalNAcβ1-4GlcNAc (LDN2) with *K*_a_ values of 9.5 × 10^4^ and 1.4 × 10^5^ M^-1^, respectively. GalNAcβ1-3Gal and GalNAcβ1-4Gal also showed high affinity for WJA (Figure 3). The alpha and beta isomers of GalNAc (GalNAc-a,b) were also good ligands for WJA, with *K*_a_ values of 1.6 × 10^4^ and 2.6 × 10^4^ M^-1^, respectively. Since approximately 70% of total sugar moieties from asialo BSM are GalNAcα-Ser/Thr [23], the data obtained from the glycan array (Figure 2) was in good agreement with that produced by FAC (Figure 3). These data obtained by FAC analysis showed that WJA bound to oligosaccharides possessing non-reducing GalNAc residues, but not Gal.

### Lectin staining of human cancer cell lines

**Figure 4:**
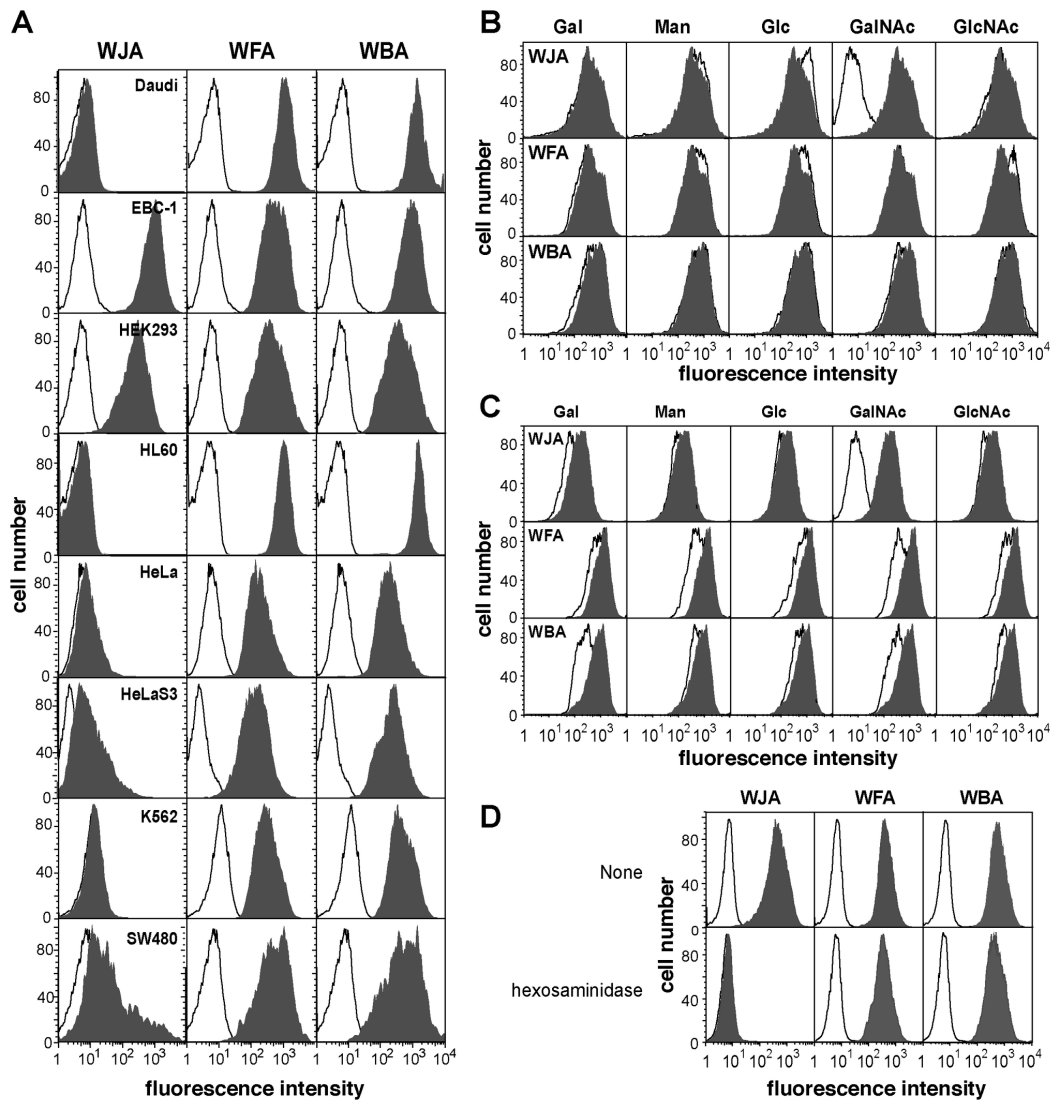
Staining of several human cancer cell lines by WJA, WFA and WBA. (A) Daudi, EBC-1, HEK293, HL60, HeLa, HeLaS3, K562, or SW480 cells were incubated with PE-labeled streptavidin alone as a control (thin line) or with 1 µg/ml biotinylated WJA, WFA or WBA followed by PE-labeled streptavidin (filled histogram) and then analyzed by flow cytometry. (B) The binding of WJA, WFA, or WBA to EBC-1 cells as measured by flow cytometry in the presence (thin line) or absence (filled histogram) of 25 mM each of the indicated monosaccharide under the same conditions described in (A). (C) The binding of WJA, WFA, or WBA to HEK293 cells was measured by flow cytometry in the presence (thin line) or absence (filled histogram) of 25 mM each of the indicated monosaccharide under the same conditions described in (A). (D) Non-treated (upper panel) or β-hexosaminidase-treated (lower panel) EBC-1 cells were incubated with WJA, WFA or WBA, respectively, and binding was analyzed by flow cytometry as described in (A) (filled histogram). Thin lines show the histogram of cells incubated with PE-labeled streptavidin alone.

 To examine the capacity of WJA to recognize cells derived from human cancerous tissues, lectin staining of several human cancer cell lines was performed and analyzed quantitatively using flow cytometry. WFA bound equally to all cell lines tested (Figure 4A), and WBA showed the same binding patterns as WFA (Figure 4A). By contrast, WJA preferentially bound to the human squamous cell carcinoma cell line, EBC-1, and human embryonic kidney-derived HEK293 cells, but not to Daudi, HL60, HeLa, HeLaS3, HepG2 or K562 cells (Figure 4A). The binding of WJA to EBC-1 and HEK293 cells was completely inhibited in the presence of 25 mM GalNAc, whereas the binding of WFA and WBA were not significantly abrogated by GalNAc (Figure 4B and Figure 4C). To assess the involvement of non-reducing terminal GalNAc residues in WJA, WFA and WBA binding to EBC-1 cells, binding of these *Wisteria* lectins to the cells was measured following β-hexosaminidase treatment of the cells. As shown in Figure 4D, WJA binding to EBC-1 cells was markedly decreased after the cells were treated with β-hexosaminidase, whereas the binding of WFA or WBA was only partially abrogated by this treatment (Figure 4D). These results strongly suggest that WJA binds specifically to terminal GalNAc residues.

### Histochemical staining of human cancerous lung tissues

**Figure 5:**
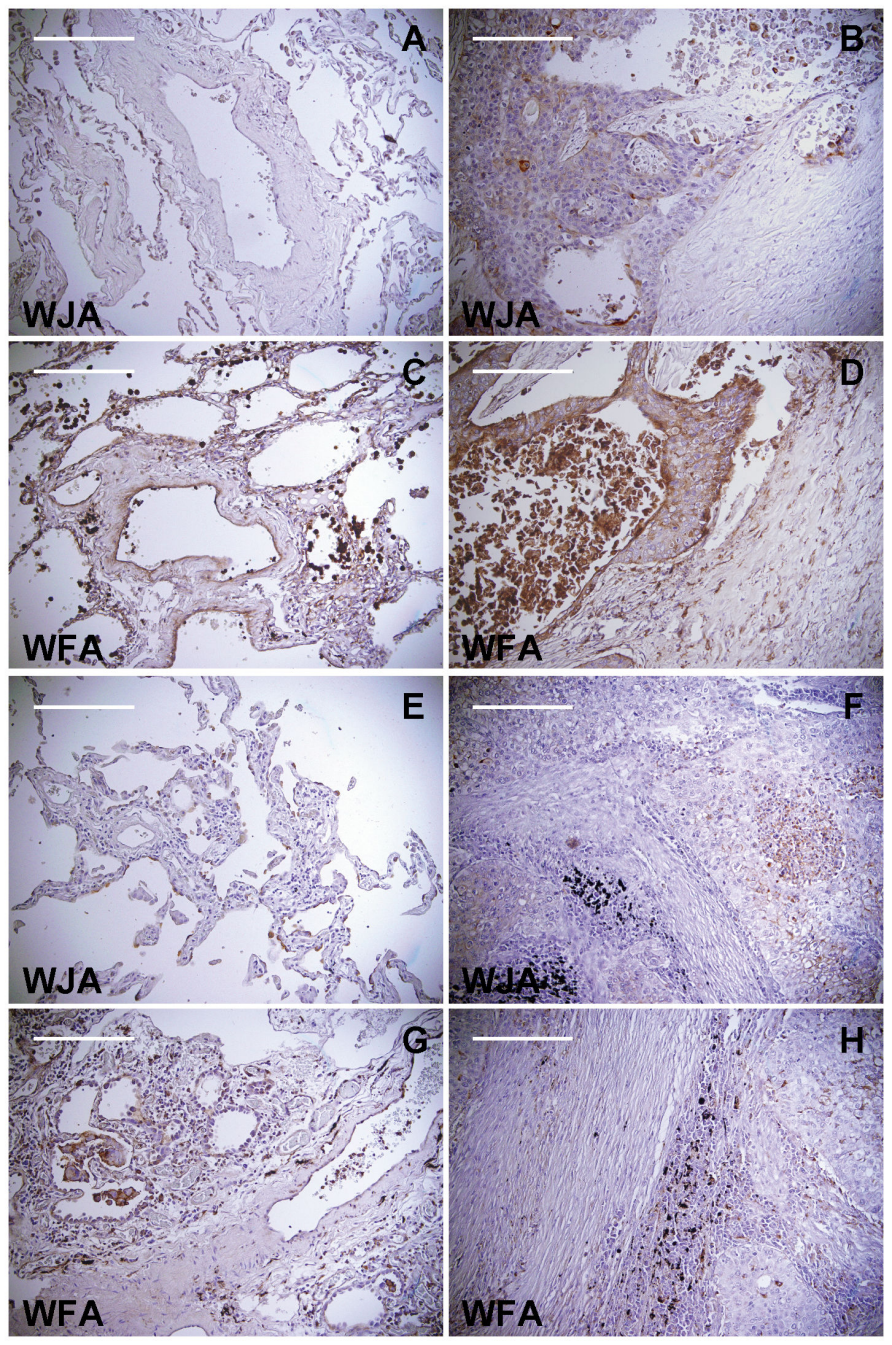
Histochemical staining of cancerous human lung tissues using WJA. Paraffin-embedded sections of human lung tissues obtained from lung squamous cell carcinomas were stained with WJA (A,B,E,F) or WFA (C,D,G,H) (brown color) and counterstained with hematoxylin. Representative data (from a 64-year-old male (A,B,C,D) and a 71-year-old male (E,F,G,H)) from 6 WJA-positive cases are shown. Cancerous portions (B,D,F,H) and normal portions (A,C,E,G) of the lung are shown. Scale bar = 200 µm.

 Because EBC-1 cells were derived from a human lung squamous cell carcinoma, we performed WJA staining of human normal and cancerous lung tissues to explore the potential utility of WJA staining for use in histochemical studies. Interestingly, the cancerous portions of lung tissue samples derived from lung squamous cell carcinoma patients distinctly reacted with WJA (Figure 5B,F), whereas normal lung tissue from the same patients were not stained by the lectin (Figure 5A,E). The cancerous area was similarly stained with WFA compared to that stained with WJA (Figure 5D,H), which may be reasonable because WFA showed the similar sugar-binding specificity compared with that of WJA. However, normal lung tissues were also stained with WFA (Figure 5C,G), which may be explained by the data showing that WFA has affinity for both terminal GalNAc and terminal Gal (Figure 2B). These results indicate that WJA may be a powerful tool for the diagnosis of human lung squamous cell carcinoma.

## Discussion

 In the present study, we purified a novel leguminous lectin with glycan-binding specificity for terminal GalNAc residues from *Wisteria japonica* seeds. *Wisteria japonica*, which had been classified as a *Millettia* species for over 100 years, was recently reclassified as a member of the genus *Wisteria* [1]. Interestingly, WJA possesses sugar-binding specificity for GalNAc, but not for Gal, which was quite different from other lectins of the *Wisteria* species, including WFA, WBA (Figure 2 and Figure 4) and *Wisteria sinensis* lectin (WSA) [24,25]. WJA strongly bound to GalNAcβ1-4GlcNAc and GalNAcβ1-3GlcNAc with a *K*_a_ of approximately 10^5^ M^-1^, GalNAcβ1-4Galβ1-4Glc and GalNAcβ1-4(NeuAcα2-3)Galβ1-4Glc with a *K*_a_ of around 4 × 10^4^ M^-1^, and the α and β isomers of GalNAc with a *K*_a_ of approximately 2 × 10^4^ M^-1^, respectively (Figure 3B). WJA is the first example of a lectin that can bind to the type I diacetyllactosamine, GalNAcβ1-3GlcNAc. In addition to binding terminal GalNAc-containing glycans, glycan array analysis revealed that unlike WJA, WFA and WBA bound to asialo α1-acid glycoproteins and agalacto α1-acid glycoproteins, whose *N*-glycans have the terminal sequences Galβ1-4GlcNAc and GlcNAcβ1-2(4)Man, respectively (Figure 2B). Other WSA also have high affinity for both terminal GalNAc and Galβ1-4GlcNAc [25], which is similar to the binding pattern exhibited by WFA and WBA, but not WJA. This is also consistent with our results showing that the hemagglutinating activities of WFA and WBA in sialidase-treated mouse erythrocytes, which possess abundant oligosaccharides possessing terminal Gal residues, were approximately 4-times stronger than that of WJA (data not shown). These findings demonstrate that WJA exhibits significant binding specificity for terminal GalNAc residues compared with other *Wisteria* lectins, including WFA, WBA and WSA.

 Terminal GalNAcβ1-4(3)GlcNAc sugar sequences are produced by β4GalNAc-T3 [12], β4GalNAc-T4 [13], and β3GalNAc-T2 [26] in humans. Expression of these *N*-acetylgalactosaminyltransferases in human tissues was quantitatively analyzed using real-time PCR. β4GalNAc-T4 is responsible for the *in vivo* synthesis of GalNAcβ1-4GlcNAc on glycoprotein hormones, such as lutropin [13]. In adult and fetal brain tissues, β4GalNAc-T4 is expressed at high levels, while β4GalNAc-T3 is not [13]. β4GalNAc-T4 is also expressed in some fetal tissues, such as kidney and lung, while β4GalNAc-T3 is expressed in the stomach, colon and testis. Furthermore, β3GalNAc-T2 is expressed in the testis, adipose tissues and skeletal muscle, although oligosaccharides having GalNAcβ1-3GlcNAc sequence have not been detected in these tissues yet [26]. Transcripts of these glycosyltransferases were specifically monitored by quantitative real-time PCR; however, their products could not monitored easily because of the lack of specific lectins or antibodies against this glycoepitope. For this reason, WJA may be a powerful tool for detecting this important epitope.

 Expression of β4GalNAc-T3 and β4GalNAc-T4 transferases in human cell lines has also been reported [12,13]. It is well known that the GalNAcβ1-4GlcNAc glycoepitope is rarely displayed on HEK293 cells [19,27], and further, that β4GalNAc-T3 and β4GalNAc-T4, which produce GalNAcβ1-4GlcNAc, are expressed in EBC-1 and HEK293 cells [12,13]. In our study, HEK293 and EBC-1 cells were stained well with WJA (Figure 4A), while Daudi and HL60 cells, which express neither β4GalNAc-T3 nor β4GalNAc-T4 [12,13], were not stained with WJA. These results demonstrate a good correlation between the expression of these GalNAc transferases and WJA binding. By contrast, WFA and WBA bound to all cells tested (Figure 4A), even though the expression levels of β4GalNAc-T3 and β4GalNAc-T4 varied widely among these cells. These results suggest that the binding of WJA is predominantly influenced by the presence of GalNAcβ1-4(3)GlcNAc, whereas WFA and WBA binding to the cells may depend on both GalNAcβ1-4(3)GlcNAc and terminal Gal-containing glycans.

 In this study, we demonstrated that WJA bound strongly to EBC-1 cells established from a human squamous cell carcinoma. Thus, tissue samples from lung squamous cell carcinoma patients were subjected to staining with WJA. Interestingly, the cancerous portions of the tissue samples stained with WJA, whereas the non-cancerous areas did not (Figure 5). Recently, it was reported that β4GalNAc-T3 enhanced malignant phenotypes of colon cancer cells [28]. Other group reported that β4GalNAc-T3 suppressed malignant phenotype of neuroblastomas by decreasing β1 integrin expression via GalNAcβ1-4GlcNAc signaling [29]. Moreover, WFA-binding proteins were reported as biomarker candidates for human cholangiocarcinoma [30,31]. Although the effect of β4GalNAc-T3 remains controversial, these results suggest that β4GalNAc-T3, β4GalNAc-T4, and/or β3GalNAc-T2 may be ectopically expressed in association with malignant transformation of squamous cells, resulting in the expression of glycans containing GalNAcβ1-4(3)GlcNAc. This hypothesis must be verified biochemically and histochemically. Further understanding of the mechanism of induced expression of these glycosyltransferases should be helpful for understanding of functional importance of GalNAcβ1-4(3)GlcNAc and the feasibility of clinical application of WJA.

## Supporting Information

Figure S1 **Structures of glycans used for glycan array analysis.** Structures of polyacrylamide-based oligosaccharides and glycoproteins used for glycan array analysis. Symbols corresponding to each monosaccharide are shown in the panel. Thin and thick bars represent alpha- and beta-linkages, respectively. Glycosidic linkage positions are shown by the numbers on the lower side of the panel.
(TIF)

Figure S2 **Glycan array analysis of Wisteria lectins.** Binding of each *Wisteria* lectin with glycans (see Figure S1) was measured by an evanescent field-activated fluorescence scanner. Oligosaccharides and glycoproteins are classified based on terminal sugar residues and indicated at the top of the upper panel.
(TIF)

Figure S3 **Structures of glycans used for frontal affinity chromatography (FAC).** Structures of PA-labeled and *p*NP-derivatized oligosaccharides used for FAC (130 total) are shown. Symbols corresponding to each monosaccharide are shown in the panel. Thin and thick bars represent alpha- and beta-linkages, respectively. Glycosidic linkage positions are shown by the numbers on the lower side of the panel.
(TIF)

Figure S4 **Frontal affinity chromatography analysis of WJA.** The *K*_a_ value of each oligosaccharide (see Figure S3) for WJA was calculated as described in Materials and Methods.
(TIF)
