# Supplementary figures and images for: Terminal N-Acetylgalactosamine-Specific Leguminous Lectin from Wisteria japonica as a Probe for Human Lung Squamous Cell Carcinoma
Source: PLoS One. 2013 Dec 13;8(12):e83886. doi: 10.1371/journal.pone.0083886 (PMC3862811; doi:10.1371/journal.pone.0083886)

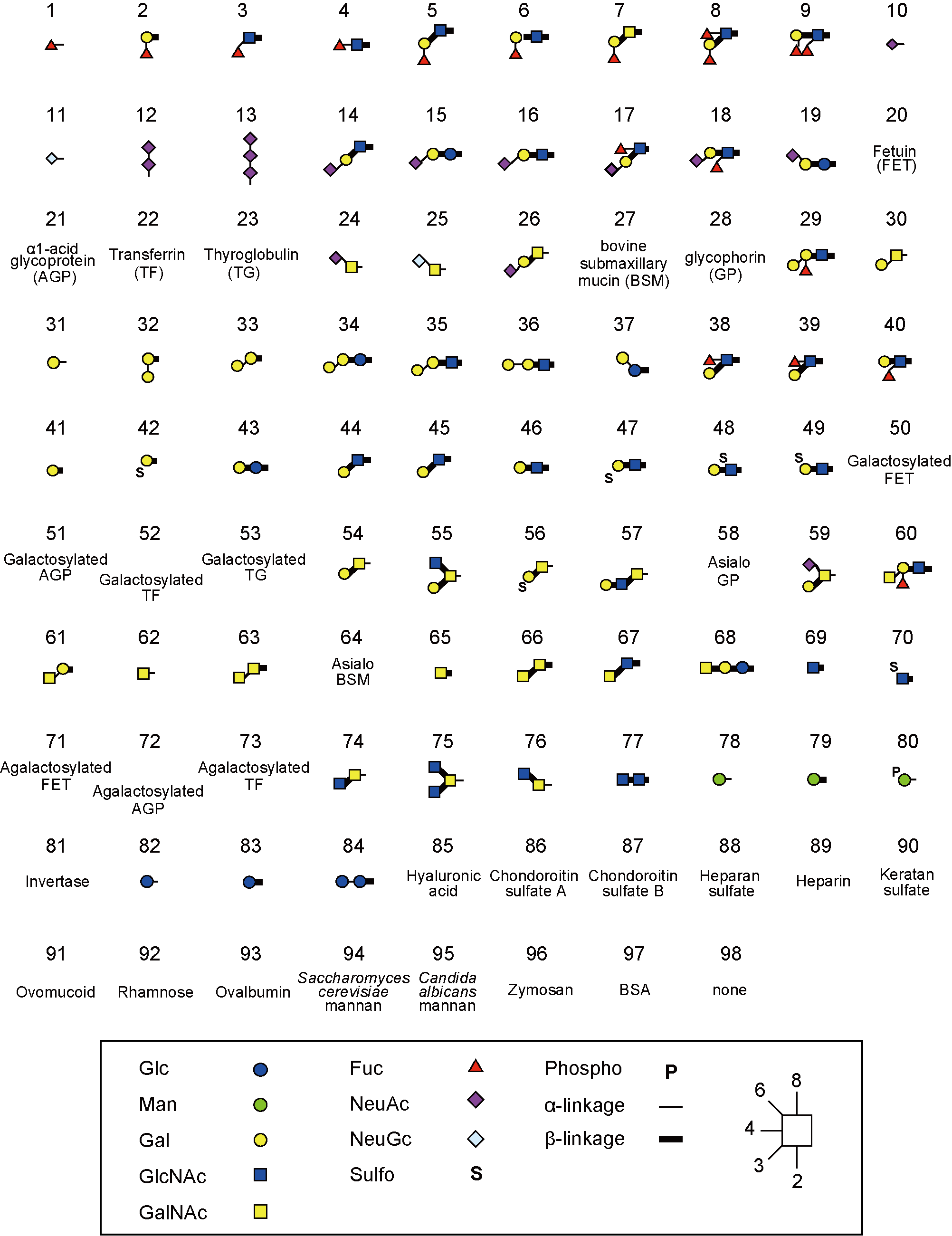

Supplement: Figure S1 — Structures of glycans used for glycan array analysis. Structures of polyacrylamide-based oligosaccharides and glycoproteins used for glycan array analysis. Symbols corresponding to each monosaccharide are shown in the panel. Thin and thick bars represent alpha- and beta-linkages, respectively. Glycosidic linkage positions are shown by the numbers on the lower side of the panel. (TIF) [file pone.0083886.s001.tif]

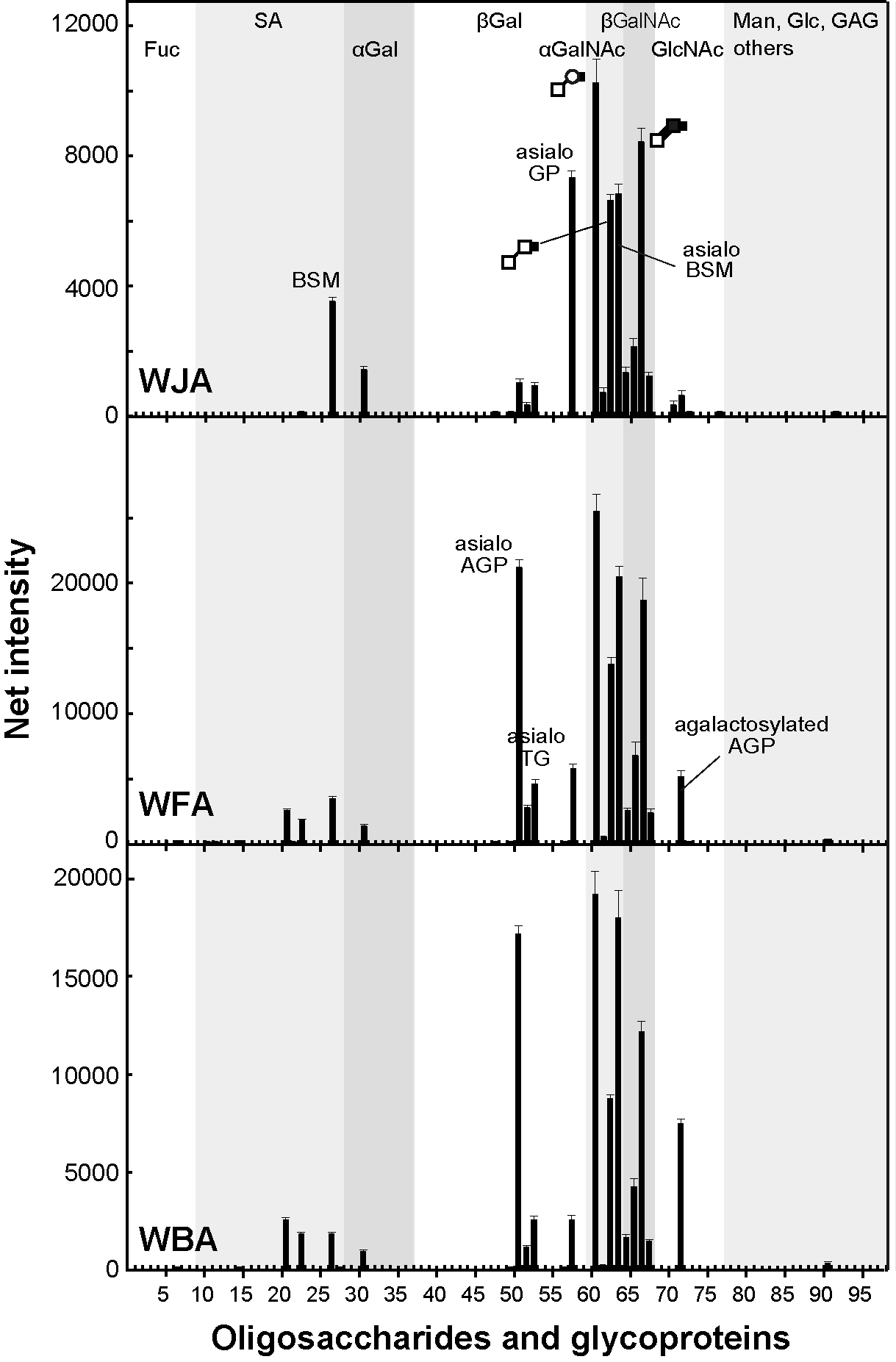

Supplement: Figure S2 — Glycan array analysis of Wisteria lectins. Binding of each Wisteria lectin with glycans (see Figure S1) was measured by an evanescent field-activated fluorescence scanner. Oligosaccharides and glycoproteins are classified based on terminal sugar residues and indicated at the top of the upper panel. (TIF) [file pone.0083886.s002.tif]

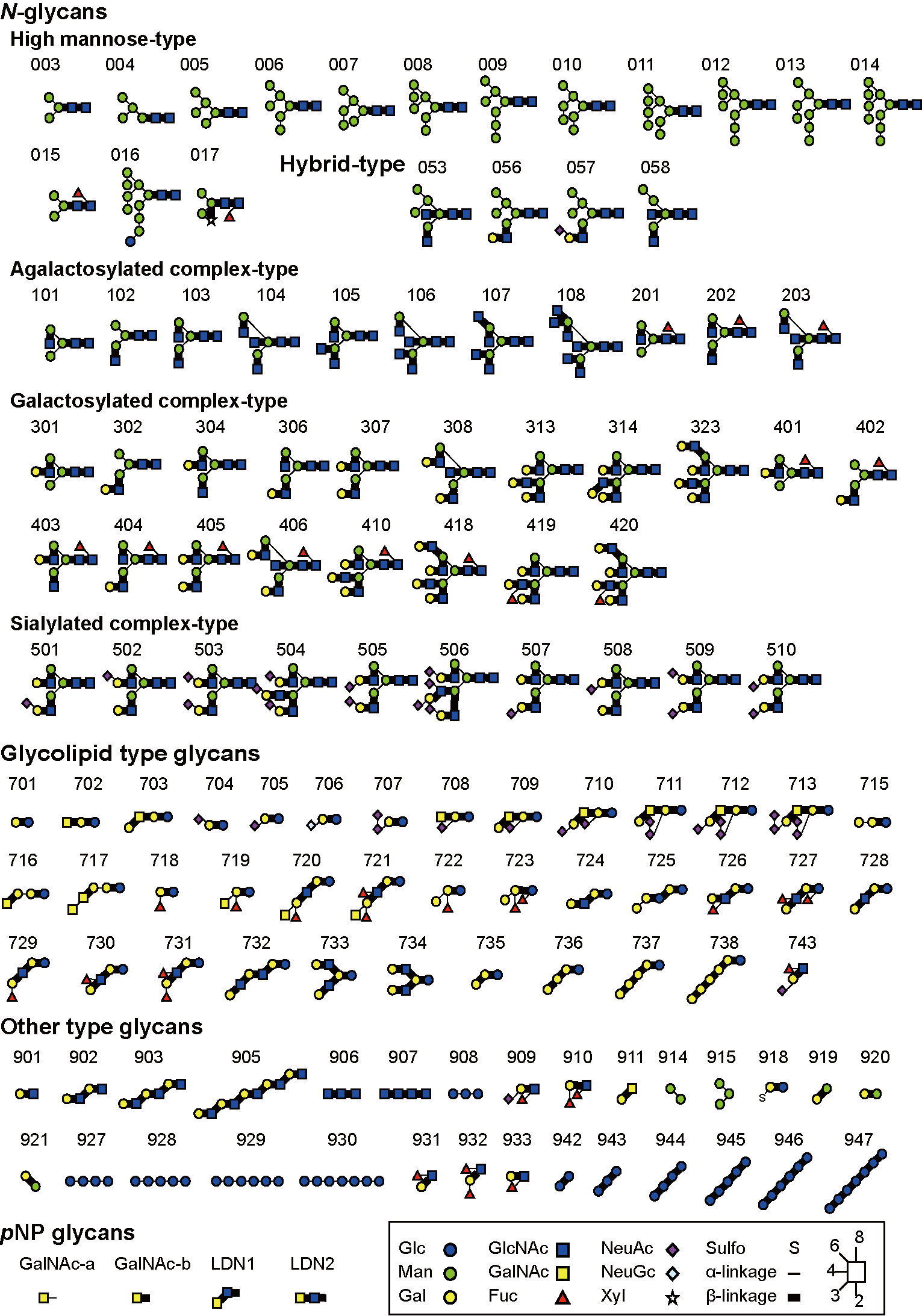

Supplement: Figure S3 — Structures of glycans used for frontal affinity chromatography (FAC). Structures of PA-labeled and pNP-derivatized oligosaccharides used for FAC (130 total) are shown. Symbols corresponding to each monosaccharide are shown in the panel. Thin and thick bars represent alpha- and beta-linkages, respectively. Glycosidic linkage positions are shown by the numbers on the lower side of the panel. (TIF) [file pone.0083886.s003.tif]

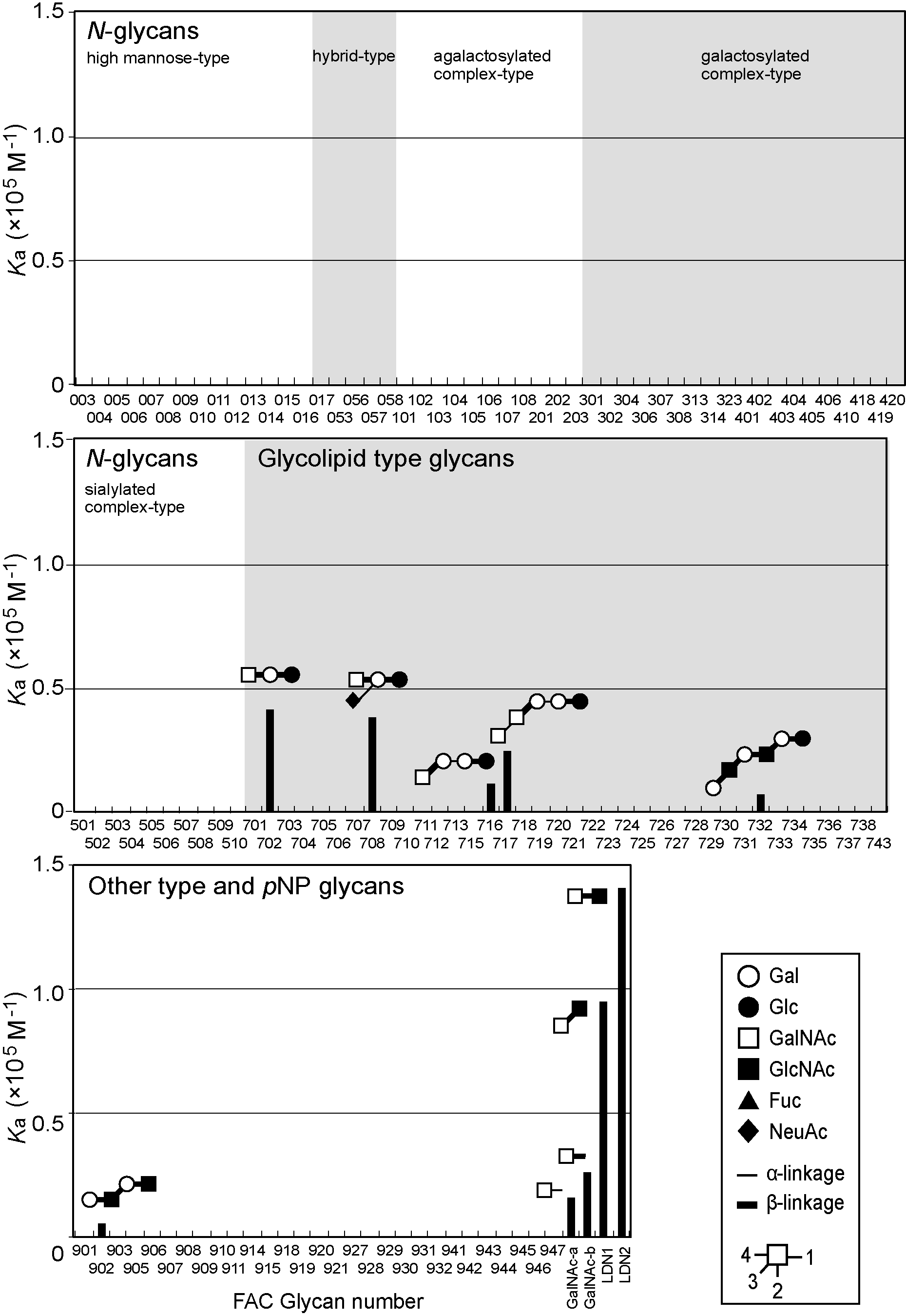

Supplement: Figure S4 — Frontal affinity chromatography analysis of WJA. The K a value of each oligosaccharide (see Figure S3) for WJA was calculated as described in Materials and Methods. (TIF) [file pone.0083886.s004.tif]
